# Author Correction: A newly identified gene *Ahed* plays essential roles in murine haematopoiesis

**DOI:** 10.1038/s41467-024-53499-5

**Published:** 2024-10-23

**Authors:** Ritsuko Nakai, Takafumi Yokota, Masahiro Tokunaga, Mikiro Takaishi, Tomomasa Yokomizo, Takao Sudo, Henyun Shi, Yoshiaki Yasumizu, Daisuke Okuzaki, Chikara Kokubu, Sachiyo Tanaka, Katsuyoshi Takaoka, Ayako Yamanishi, Junko Yoshida, Hitomi Watanabe, Gen Kondoh, Kyoji Horie, Naoki Hosen, Shigetoshi Sano, Junji Takeda

**Affiliations:** 1 Department of Haematology and Oncology, Graduate School of Medicine, Osaka University, Suita, Osaka 565-0871 Japan; 2 Department of Haematology, Osaka International Cancer Institute, Osaka, Osaka 541-8567 Japan; 3 Department of Haematology, Suita Municipal Hospital, Suita, Osaka 564-0018 Japan; 4 Department of Genome Biology, Graduate School of Medicine, Osaka University, Suita, Osaka 565-0871 Japan; 5 Department of Dermatology, Kochi Medical School, Kochi University, Nankoku, Kochi 783-8505 Japan; 6 Department of Microscopic and Developmental Anatomy, Tokyo Women’s Medical University, Shinjuku-ku, Tokyo 162-8666 Japan; 7 Department of Haematology, National Hospital Organisation Osaka National Hospital, Osaka, Osaka 540-0006 Japan; 8 Department of Experimental Immunology, Immunology Frontier Research Centre, Osaka University, Suita, Osaka 565-0871 Japan; 9 Integrated Frontier Research for Medical Science Division, Institute for Open and Transdisciplinary Research Initiatives, Osaka University, Suita, Osaka 565-0871 Japan; 10 Genome Information Research Centre, Research Institute for Microbial Diseases, Osaka University, Suita, Osaka 565-0871 Japan; 11 Developmental Genetics Group, Graduate School of Frontier Biosciences, Osaka University, Suita, Osaka 565-0871 Japan; 12 Department of Physiology II, Nara Medical University, Kashihara, Nara 634-8521 Japan; 13 Laboratory of Animal Experiments for Regeneration, Institute for Frontier Life and Medical Sciences, Kyoto University, Kyoto, Kyoto 606-8507 Japan; 14 Laboratory of Cellular Immunotherapy, World Premier International Immunology Frontier Research Centre, Osaka University, Suita, Osaka 565-0871 Japan; 15 Research Institute for Microbial Diseases, Osaka University, Suita, Osaka 565-0871 Japan

**Keywords:** Lymphopoiesis, Development, Haematopoietic stem cells, Differentiation

Correction to: *Nature Communications* 10.1038/s41467-024-49252-7, published online 25 June 2024

The original version of this Article contained an error in the schematic representation of the AHED protein in Fig. 2C. This error has been corrected in both the PDF and HTML versions of the Article.

