# Supplementary material for: A newly identified gene Ahed plays essential roles in murine haematopoiesis
Source: Nat Commun. 2024 Jun 25;15:5090. doi: 10.1038/s41467-024-49252-7 (PMC11199565; doi:10.1038/s41467-024-49252-7)
Supplement: Supplementary file 1 — Supplementary Information [file 41467_2024_49252_MOESM1_ESM.pdf]

Supplementary Information

A newly identified gene *Ahed* plays essential roles in murine haematopoiesis

Ritsuko Nakai[1], Takafumi Yokota[1,2,*], Masahiro Tokunaga[3,4], Mikiro Takaishi[5], Tomomasa Yokomizo[6], Takao Sudo[1,7], Henyun Shi[1], Yoshiaki Yasumizu[8,9], Daisuke Okuzaki[9,10], Chikara Kokubu[4], Sachiyo Tanaka[4], Katsuyoshi Takaoka[11], Ayako Yamanishi[4], Junko Yoshida[4,12], Hitomi Watanabe[13], Gen Kondoh[13], Kyoji Horie[4,12], Naoki Hosen[1,9,14], Shigetoshi Sano[5], and Junji Takeda[15,*]

[1]Department of Haematology and Oncology, Graduate School of Medicine, Osaka University, Suita, Osaka, Japan.

[2]Department of Haematology, Osaka International Cancer Institute, Osaka, Osaka, Japan.

[3]Department of Haematology, Suita Municipal Hospital, Suita, Osaka, Japan.

[4]Department of Genome Biology, Graduate School of Medicine, Osaka University, Suita, Osaka, Japan.

[5]Department of Dermatology, Kochi Medical School, Kochi University, Nankoku, Kochi, Japan.

[6]Department of Microscopic and Developmental Anatomy, Tokyo Women's Medical University, Tokyo, Japan.

[7]Department of Haematology, National Hospital Organisation Osaka National Hospital, Osaka, Osaka, Japan.

[8]Department of Experimental Immunology, Immunology Frontier Research Centre, Osaka University, Suita, Osaka, Japan.

[9]Integrated Frontier Research for Medical Science Division, Institute for Open and Transdisciplinary Research Initiatives, Osaka University, Suita, Osaka, Japan.

[10]Genome Information Research Centre, Research Institute for Microbial Diseases, Osaka University, Suita, Osaka, Japan.

[11]Developmental Genetics Group, Graduate School of Frontier Biosciences, Osaka University, Suita, Osaka, Japan.

[12]Department of Physiology II, Nara Medical University, Kashihara, Nara, Japan.

[13]Laboratory of Animal Experiments for Regeneration, Institute for Frontier Life and Medical Sciences, Kyoto University, Kyoto, Kyoto, Japan.

[14]Laboratory of Cellular Immunotherapy, World Premier International Immunology Frontier Research Center, Osaka University, Suita, Osaka, Japan.

[15]Research Institute for Microbial Diseases, Osaka University, Suita, Osaka, Japan.

This PDF contains Supplementary Figures 1-8 and Supplementary Tables 1-9.

Supplementary Figure 1

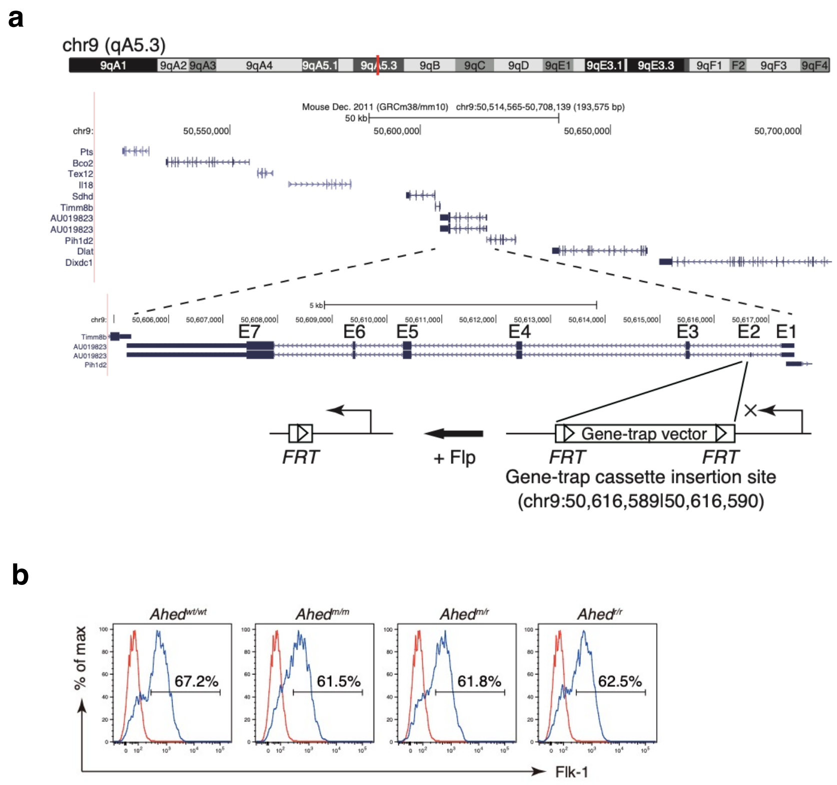

Supplementary Fig. 1 | Homozygous mutant ESCs screening identified *Ahed*. a UCSC genome browser screenshot of the mouse *AU019823* locus (upper) and the insertion site of the retrovirus-based gene-trap cassettes (lower). Both ends of the cassette are flanked by *FRT* sites (open triangles), allowing removal of the cassette using Flp recombinase. Coordinates on chromosome 9 are based on the GRCm38/mm10 mouse assembly. *AU019823* gene has two transcript variants, both of which encode the same protein. Chr, chromosome; E, exon. **b** Representative flow cytometric analysis of Flk-1^{+} cells on day 4 after the induction of ESC differentiation, showing no difference between the four genetic backgrounds (*Ahed*$^{wt/wt}$, parental wild-type ESCs; *Ahed*$^{m/m}$, homozygous gene-trap mutant ESCs; *Ahed*$^{m/r}$, heterozygous revertant ESCs; *Ahed*$^{r/r}$, homozygous revertant ESCs).

Supplementary Figure 2

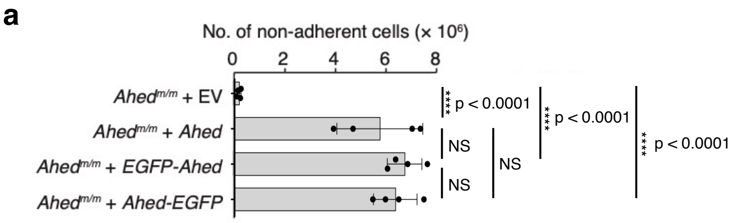

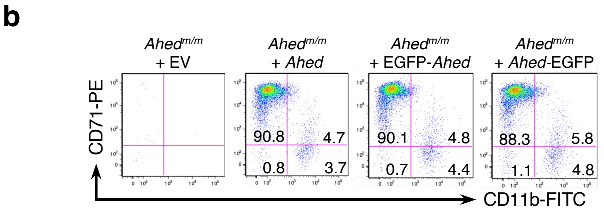

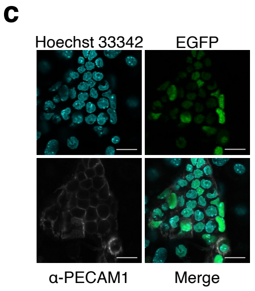

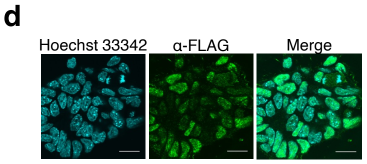

Supplementary Fig. 2 | The *Ahed* gene product is a nuclear protein. a The number of haematopoietic cells on day 13 ($n = 4$) of *in vitro* differentiation. *Ahed*$^{m/m}$ ESCs expressing each plasmid were subjected to haematopoietic differentiation by co-culturing on OP9 stroma. EV, empty vector. **b** Flow cytometric analysis of haematopoietic cells harvested on day 13 of each culture. Percentages of cells in each quadrant are shown. **c** Confocal microscopy of PECAM1+ endothelial cells differentiated from *EGFP-Ahed*-expressing *Ahed*$^{m/m}$ ESCs. After 8-day culture with OP9, cells were stained for PECAM1. Nuclei were counterstained with Hoechst 33342. Scale bars, 20 µm. **d** Immunofluorescence analysis of *Ahed*$^{m/m}$ ESCs expressing *FLAG*-tagged full-length *Ahed* cDNA. ESCs were stained with anti-FLAG Ab. Nuclei were counterstained with Hoechst 33342. Scale bars, 20 µm. Data are presented as mean ± s.d. Statistical significance in **a** was determined by one-way ANOVA with Brown-Forsythe test. ****$p < 0.0001$; NS, not significant. Source data are provided as a Source Data file.

Supplementary Figure 3

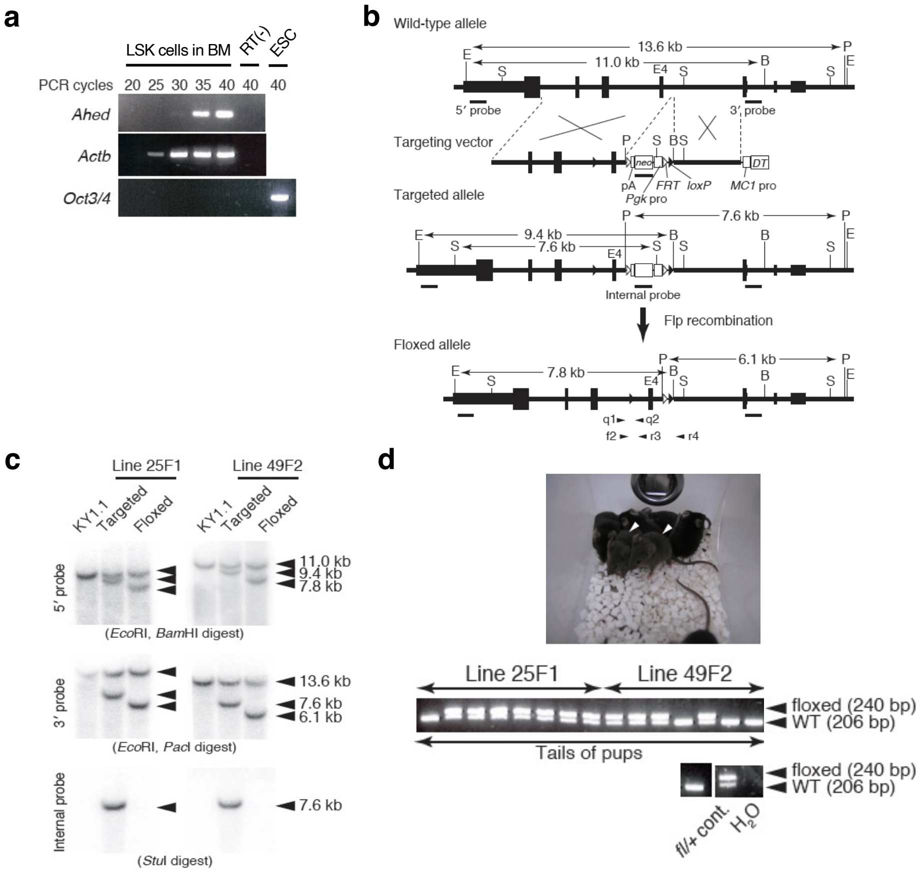

e

	Ahed$^{+/+}$	*Ahed*$^{fl/+}$	*Ahed*$^{fl/fl}$	*P*-value
WBC	1.24 ± 0.17	1.17 ± 0.39	1.27 ± 0.21	0.78
RBC	777 ± 142	729 ± 89	788 ± 60	0.51
Plt	65.4 ± 13.8	58.2 ± 12.0	55.4 ± 11.9	0.31

f

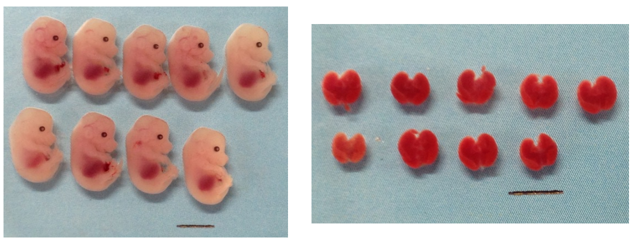

Supplementary Fig. 3 | *Ahed* deletion causes severely anaemic and embryonic demise. a The expression level of *Ahed* in the haematopoietic stem/progenitor fraction in adult bone marrow (BM) was analysed by semiquantitative reverse transcription PCR. The *Actb* gene was used as positive control and *Oct3/4* was used as negative control. LSK, Lineage$^-$Sca1$^+$c-kit^{high}; RT, reverse transcription; ESC indicates the expression level of *Oct3/4* in ESCs. **b** Conditional targeting strategy of the *Ahed* locus. The locations of probes for Southern blotting are shown. The genotyping primer pair q1 and q2, and the primer trio f2, r3, and r4 are also denoted. B, *Bam*HI; E, *Eco*RI; P, *Pac*I; S, *Stu*I; *MC1* pro, *MC1* promoter; *Pgk* pro, *Pgk1* promoter; *neo*, *neo* positive selection cassette; pA, poly-A; *DT*, *diphtheria toxin* negative selection cassette. **c** Southern blot analyses of parental and targeted ESCs. **d** Pups with agouti coat color (upper, white arrowheads) generated by mating a high contribution male chimera of line 49F2 to a C57BL/6J female mouse, indicating germline transmission. Genotyping results of F1 pups using primers q1 and q2 (lower). We eventually produced two lines of cKO mice (25F1 and 49F2), both of which presented the same haematological phenotype. Consequently, almost all of the experiments were performed using line 25F1 mice. **e** Peripheral blood counts ($\times$ 10^{10}/L) of 6-week-old male *Ahed*$^{+/+}$ (n = 7), *Ahed*$^{fl/+}$ (n = 7), and *Ahed*$^{fl/fl}$ (n = 8) mice. WBC; white blood cells, RBC; red blood cells, Plt; platelets. **f** Embryos and their foetal livers recovered at E14.5. Arrows indicate *Ahed* cKO (*Vav1-cre Ahed*$^{fl/fl}$) genotype carriers. Scale bars, 5 mm. Data are presented as mean $\pm$ s.d. Statistical significance in **e** was determined by False-discovery-rate-corrected with the Benjamini and Hochberg method. Source data are provided as a Source Data file.

Supplementary Figure 4

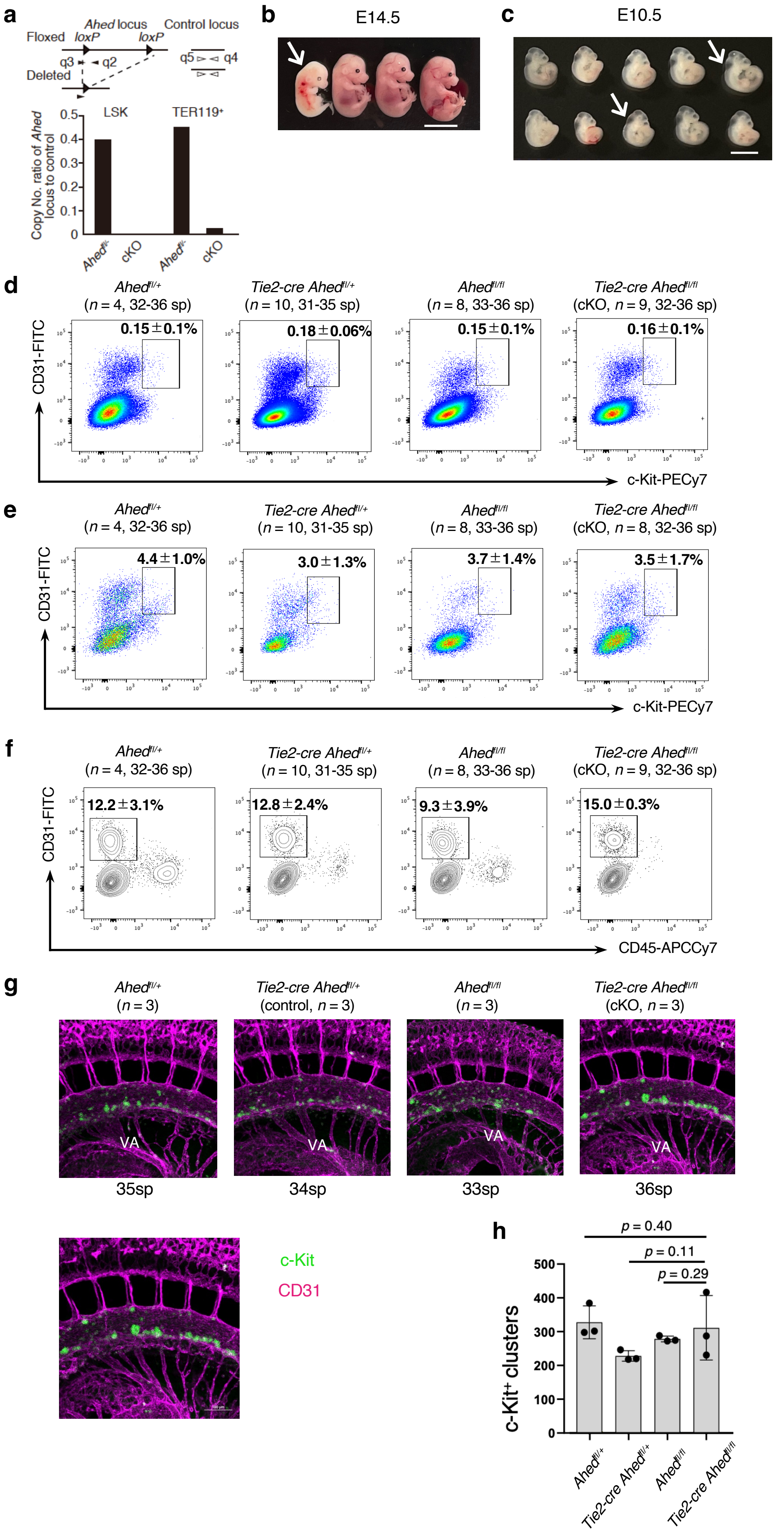

Supplementary Fig. 4 | *Ahed* is unessential for endothelial-to-haematopoietic transition. a Schematic strategy to estimate the efficiency of Cre/*loxP* recombination (upper). The copy number of the floxed segment was compared with that of the control segment using real-time qPCR. Depicted are primers q2 and q3 (black arrowheads) for the floxed segment and q4 and q5 (open arrowheads) for the control segment. Data are shown as the means of two embryos of each genotype (lower). **b** Embryos recovered at E14.5. White arrowheads indicate the *Ahed* cKO (*Tie2-cre Ahed$^{fl/fl}$*) embryos. Scale bar, 5 mm. **c** Embryos recovered at E10.5. White arrowheads indicate the *Ahed* cKO (*Tie2-cre Ahed$^{fl/fl}$*) embryos. Scale bar, 2 mm. **d-f** FACS analysis of the caudal half region containing vitelline artery (VA) and umbilical artery (UA) (**d,f**) or yolk sac (**e**) at E10.5 from *Ahed$^{fl/+}$, Tie2-cre Ahed$^{fl/+}$*, *Ahed$^{fl/fl}$, Tie2-cre Ahed$^{fl/fl}$* (cKO) embryos. Each subset group was defined as follows; c-Kit$^+$CD31$^+$; haematopoietic cluster (**d,e**) and c-Kit$^+$CD31$^+$CD45$^-$; haemogenic endothelial cells (**f**). Numbers indicate the percentage of each fraction. Results shown are representative of three independent experiments. **g** Whole-mount immunostaining of E10.5 embryo for c-Kit and CD31 expression; *Ahed$^{fl/+}$* (32-36 sp), *Tie2-cre Ahed$^{fl/+}$* (31-35 sp), *Ahed$^{fl/fl}$* (33-36 sp), and *Tie2-cre Ahed$^{fl/fl}$* (cKO) (32-36 sp). VA, vitelline artery. The scale bar is shown in the lower diagram. Scale bar: 100µm. **h** Graph shows the number of c-Kit$^+$ cells in the middle area (7 somites long) of the dorsal aorta (DA) from *Ahed$^{fl/+}$* (E10.5, 34-35 sp), *Tie2-cre Ahed$^{fl/+}$* (E10.5, 34 sp), *Ahed$^{fl/fl}$* (E10.5, 33-36 sp), and *Tie2-cre Ahed$^{fl/fl}$* (cKO) (E10.5, 33-36 sp); *n* = 3. Data are presented as mean ± s.d. Statistical significance in **d**-**f**,**h** was determined by one-way ANOVA with Brown-Forsythe test. Source data are provided as a Source Data file.

Supplementary Figure 5

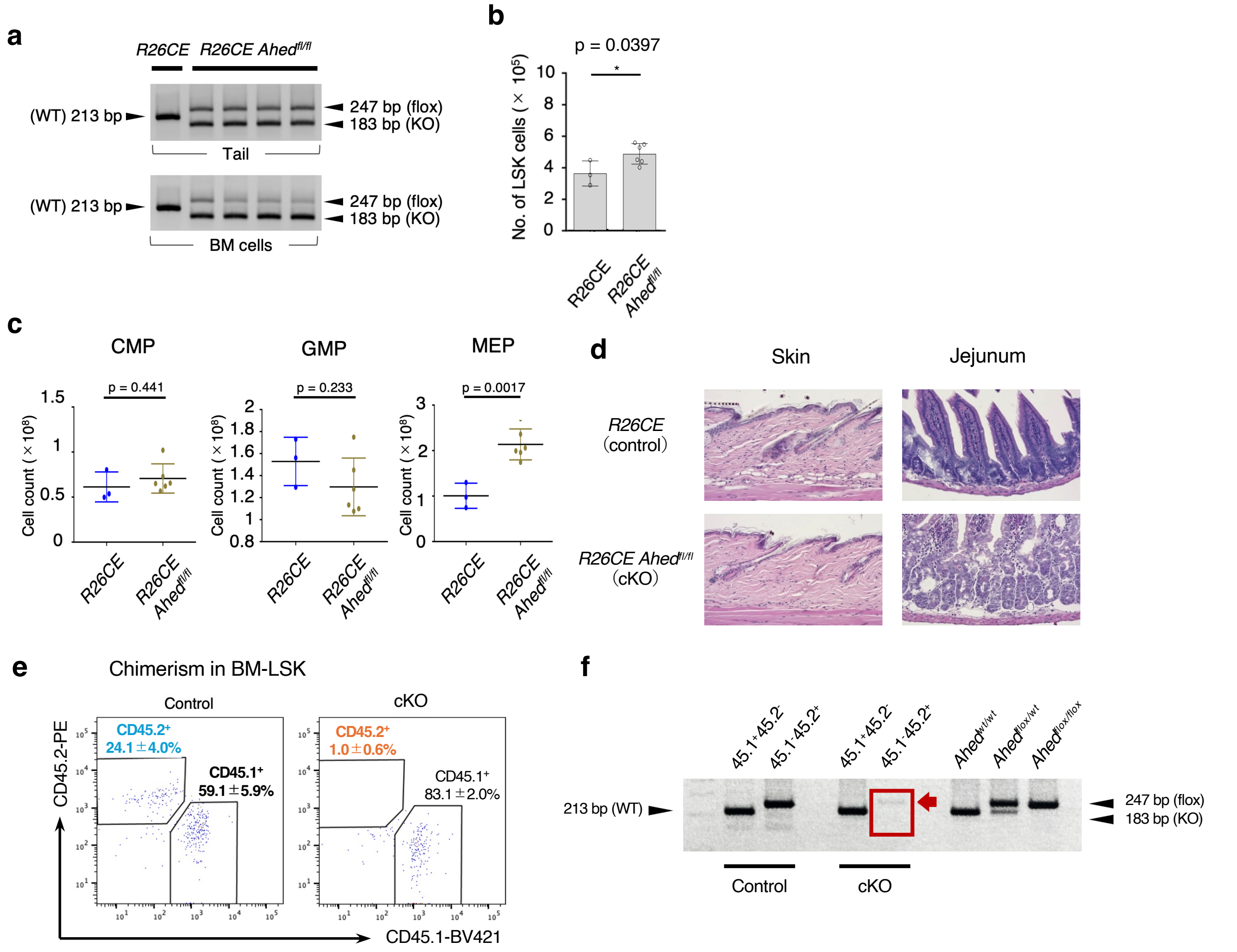

Supplementary Fig. 5 | *Ahed* plays essential roles in adult haematopoiesis. a Genotyping results of tail and BM cells from *R26CE* and *R26CE* $Ahed^{fl/fl}$ mice, resulting in the efficient deletion of the *Ahed* gene in the BM cells of KO mice. **b** Absolute numbers of LSK cells from BM; $n = 3$ in *R26CE* (control) and $n = 6$ in *R26CE* $Ahed^{fl/fl}$ (KO). n.s.; no significance. **c** The number of total CMPs, GMPs, and MEPs; $n = 3$ in *R26CE* (control) and $n = 6$ in *R26CE* $Ahed^{fl/fl}$ (KO). **d** H&E staining of skin and jejunum tissues from *R26CE* and *R26CE* $Ahed^{fl/fl}$ mice. **e** Chimerism of LSK in recipients' bone marrow. Numbers in each figure indicate percentages of each fraction. **f** PCR examinations detected incompletely deleted $Ahed^{fl/fl}$ band (247 bp) in the small CD45.2^{+} population reconstituted from the 4-OHT-treated *R26CE* $Ahed^{fl/fl}$ LSK cells. Data are presented as mean ± s.d. Statistical significance in **b**,**c** was determined by two-sided unpaired Student's *t*-test, and in **e** by one-way ANOVA with Brown-Forsythe test. $^{*}p < 0.05$. NS, not significant. Source data are provided as a Source Data file.

Supplementary Figure 6

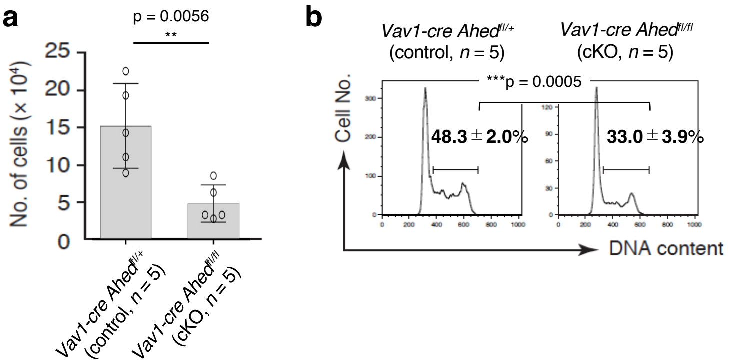

Supplementary Fig. 6 | *Ahed* deficiency induces cellular apoptosis. a Number of cells yielded on day 3 culture of the FL-derived LSK cells. **b** DNA content of cultured cells was examined on day 3 culture of the FL-derived LSK cells, using propidium iodide (PI) staining. Data are presented as mean ± s.d. Statistical significance in **a**,**b** was determined by two-sided unpaired Student's *t*-test. **$p < 0.01$, ***$p < 0.001$. Source data are provided as a Source Data file.

Supplementary Figure 7

a

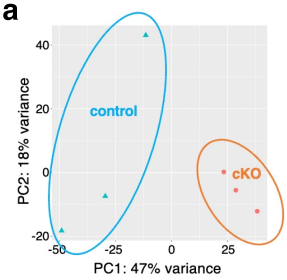

b

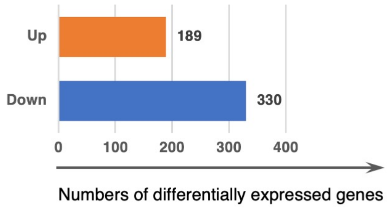

c

Direction	Adj.Pval	nGenes	Pathways
Up-regulated	5.4e-10	56	Circulatory system development
	9.2e-10	44	Vasculature development
	9.2e-10	40	Blood vessel morphogenesis
	1.9e-09	36	Angiogenesis
	1.9e-09	42	Blood vessel development
	4.6e-09	88	Anatomical structure morphogenesis
	1.1e-08	45	Tube morphogenesis
	2.9e-08	53	Cell adhesion
	8.1e-08	97	Regulation of multicellular organismal process
	8.5e-08	49	Anatomical structure formation involved in morphogenesis
	2.0e-07	64	Locomotion
	2.2e-07	124	System development
	2.2e-07	46	Regulation of locomotion

Direction	Adj.Pval	nGenes	Pathways
Down-regulated	3.3e-46	75	Defense response to other organism
	2.4e-45	67	Innate immune response
	1.8e-43	82	Response to external biotic stimulus
	1.4e-42	84	Defense response
	5.1e-40	30	Response to interferon-beta
	7.4e-40	84	Interspecies interaction between organisms
	4.8e-37	77	Immune response
	9.6e-36	26	Cellular response to interferon-beta
	4.5e-34	95	Immune system process
	4.1e-30	60	Response to cytokine
	2.1e-29	40	Response to virus
	5.3e-28	88	Response to external stimulus
	2.4e-26	34	Defense response to virus

d

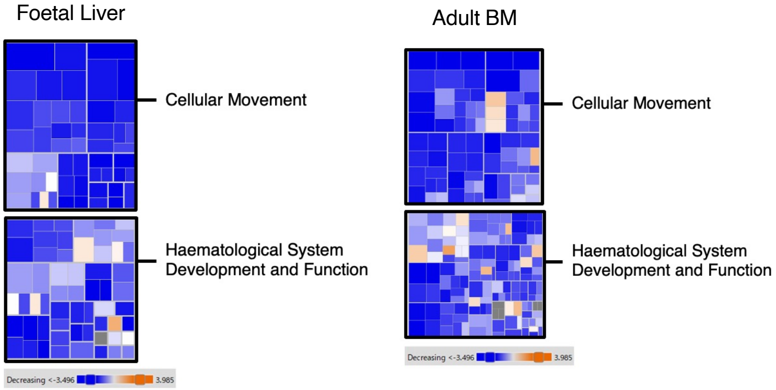

Supplementary Fig. 7 | *Ahed* deficiency affects the expression of multiple genes. a Principal component analysis results. The figure displays a scatter plot of the two independent principal components (PCs) of the data. Each point represents an RNA-Seq sample; *Vav1-cre Ahed*$^{fl/+}$ (control) and *Vav1-cre Ahed*$^{fl/fl}$ (cKO) are well-separated in PC1. **b** The number of differentially expressed genes from RNA-seq in LSK cells from *Vav1-cre Ahed*$^{fl/fl}$ (cKO) foetal liver were compared with those from *Vav1-cre Ahed*$^{fl/+}$ (control) foetal liver. **c** Pathway analysis with iDEP.96, respectively up-regulated and down-regulated, in LSK cells from *Vav1-cre Ahed*$^{fl/fl}$ (cKO) foetal liver were compared with those from *Vav1-cre Ahed*$^{fl/+}$ (control) foetal liver. Adj.Pval; adjusted p-value, nGenes; the number of genes. **d** Color-coded heat map analyses regarding "Cellular movement" and "Haematological System Development and Function" among enriched biological functional categories in E14.5 FL (left) and BM (right).

Supplementary Figure 8

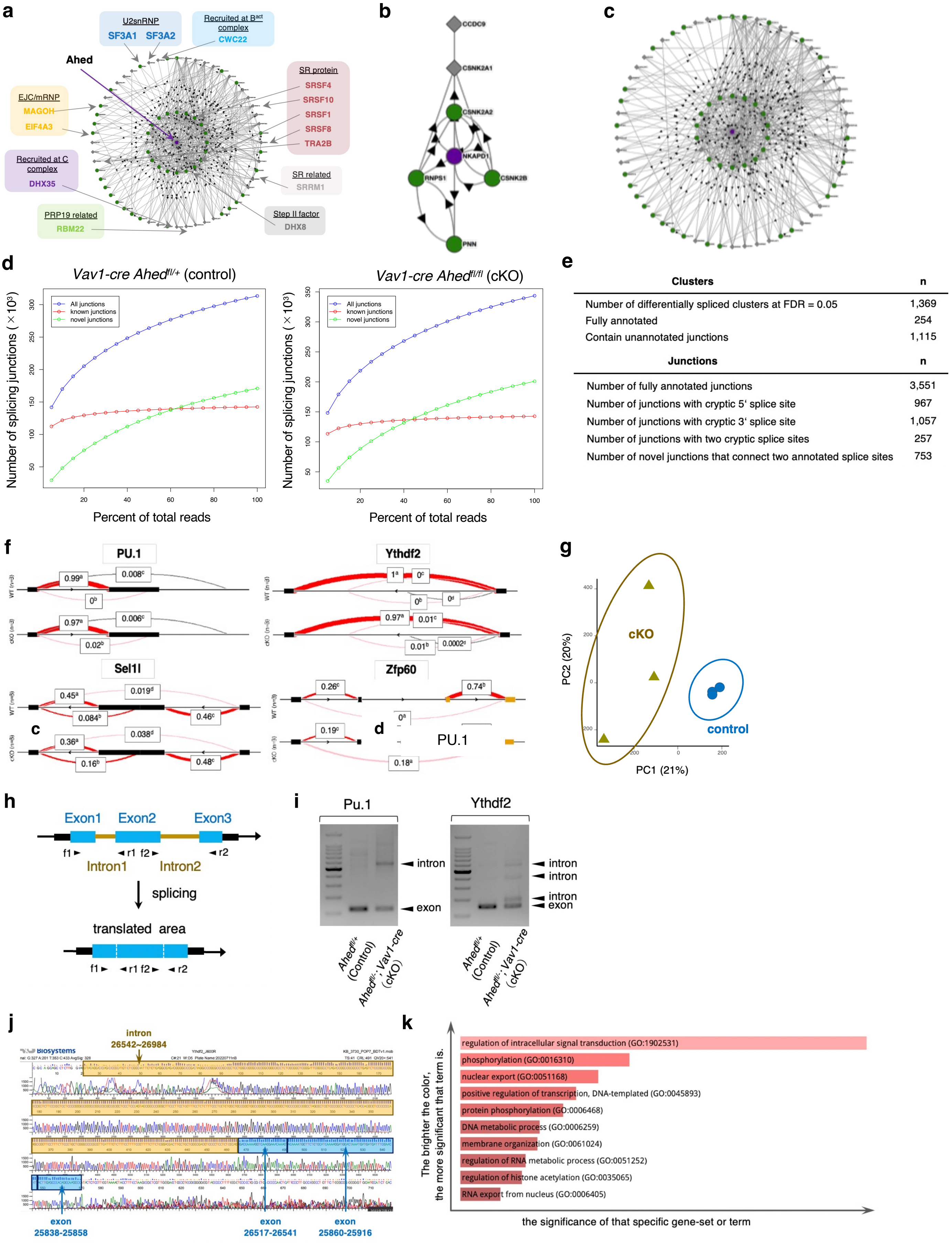

Clusters	n
Number of differentially spliced clusters at FDR = 0.05	1,369
Fully annotated	254
Contain unannotated junctions	1,115
**Junctions**	**n**
Number of fully annotated junctions	3,551
Number of junctions with cryptic 5' splice site	967
Number of junctions with cryptic 3' splice site	1,057
Number of junctions with two cryptic splice sites	257
Number of novel junctions that connect two annotated splice sites	753

Supplementary Fig. 8 | *Ahed* deficiency induces abnormal transcripts of critical genes for haematopoiesis. a Visualisation using the biophysical interactions of ORFeome-based complexes 3.0 (BioPlex 3.0) network database indicated a close correlation of the Ahed protein with numerous RNA splicing related proteins. Combined data obtained from HEK293T (human renal cancer cell line) and HCT116 (human colon cancer cells). **b, c** Visualization using the biophysical interactions of ORFeome-based complexes 3.0 (BioPlex 3.0) network database in HEK293T (human renal cancer cell line) (**b**) and HCT116 (human colon cancer cells) (**c**), respectively. **d** Saturation plots for the number of splicing junctions detected in each sample (left; *Vav1-cre Ahed*$^{fl/+}$ (control), right; *Vav1-cre Ahed*$^{fl/fl}$ (cKO)). **E** Summary of results on the number of clusters and junctions detected by Leafcutter. There are numerous differentially spliced clusters. The detection power is also stronger due to the deeper interpretation of the sequence. **f** Splicing event visualisation in four representative genes, *PU.1*, *Ythdf2*, *Sel1l*, and *Zfp60* involved in haematopoietic stem cells through the application of Leafcutter. Summary of results on the number of clusters and junctions detected by Leafcutter. There are numerous differentially spliced clusters. The detection power is also stronger due to the deeper interpretation of the sequence. **g** Principal component analysis results. The figure displays a scatter plot of the two independent principal components (PCs) of the data. Each point represents an RNA-Seq sample; *Vav1-cre Ahed*$^{fl/+}$ (control) and *Vav1-cre Ahed*$^{fl/fl}$ (cKO) are well-separated in PC1. **h** Schematic diagram of specific primer design to examine splicing and aberrant transcripts by conventional reverse transcription PCR (RT-PCR). **i** Electrophoresis results of RT-PCR with each specific primer (Supplementary Table 5). Results shown are representative of two genes. **j** Intron sequences extracted from gels of long PCR products and identified by Sanger sequencing. Result shown is representative of a gene *Ythdf2*. **k** Enriched pathways in genes (intron clusters with FDR < 0.05 and mapped to a single gene) by Enrichr.

Supplementary Table 1

Supplementary Table 1. Mutant ES cell clones examined in this study.

Gene Symbol	Chromosome	Gene Name	Phenotypes of knockout mice*
Nr5a2	1	nuclear receptor subfamily 5, group A, member 2	die around E7.5
Phf20	2	PHD finger protein 20	multiple phenotype
Fubp3	2	far upstream element (FUSE) binding protein 3	not reported
Cdca7	2	cell division cycle associated 7	not reported
Csnk2a1	2	casein kinase 2, alpha 1 polypeptide	die by E11.5
Gpatch4	3	G patch domain containing 4	not reported
Cdc42se1	3	CDC42 small effector 1	not reported
Fryl	5	furry homolog-like (Drosophila)	embryonic or preweaning lethality
G3bp2	5	GTPase activating protein (SH3 domain) binding protein 2	preweaning lethality
Kntc1	5	kinetochore associated 1	kinked tail
Gnb2	5	guanine nucleotide binding protein, beta 2	not reported
Lrch4	5	leucine-rich repeats and calponin homology (CH) domain containing 4	not reported
Lnx2	5	ligand of numb-protein X 2	not reported
Manf	9	mesencephalic astrocyte-derived neurotrophic factor	diabetes
Nedd4	9	neural precursor cell expressed, developmentally down-regulted gene 4	neonatal lethality
AU019823	9	expressed sequence AU019823	not reported
Igsf9b	9	immunoglobulin superfamily, member 9B	not reported
Msantd2	9	Myb/SANT-like DNA-binding domain containing 2	not reported
Leo1	9	Leo1, Paf1/RNA polymerase II complex component, homolog (S. cerevisiae)	not reported
Tbrg4	11	transforming growth factor beta regulated gene 4	not reported
Nmt1	11	N-myristoyltransferase 1	die between E3.5 an E7.5

* Phenotypes based on the MGI website (http://www.informatics.jax.org).

Supplementary Table 2

Supplementary Table 2. *Ahed* expression for each tissue in BioGPS database.

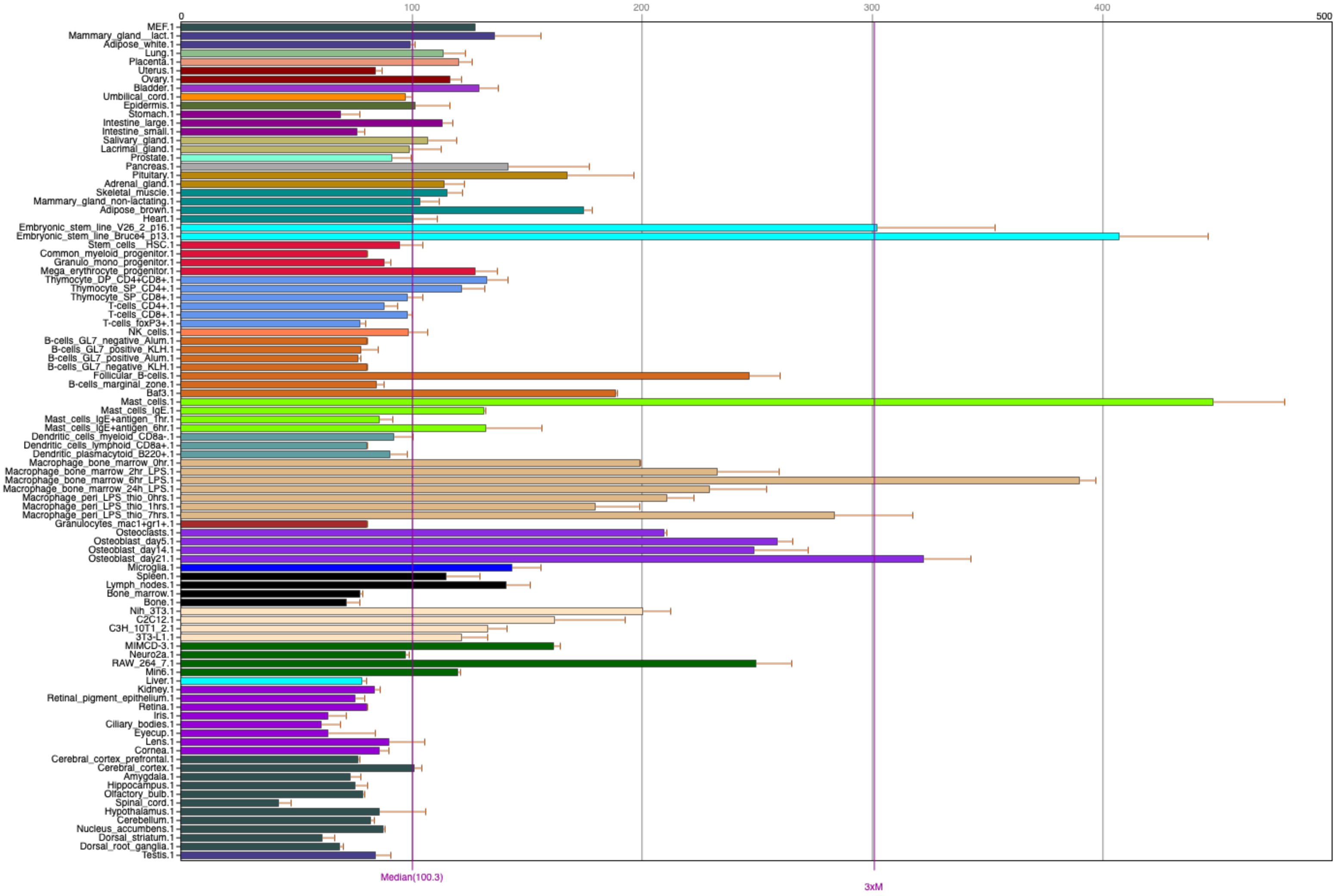

Supplementary Table 3

Supplementary Table 3. Primers used for reverse transcription or real-time PCR to investigate expression levels of HSC-related genes in E14.5 FL LSK cells in *Ahed* cKO mice.

Primer Name	Gene	Sequence (5'-3')
Gapdh-F*	*Gapdh*	CATCACTGCCACCCAGAAGACTG
Gapdh-R*	*Gapdh*	ATGCCAGTGAGCTTCCCGTTCAG
Actb-F*	*Actb*	CAGGGTGTGATGGTGGGAATGGGTCAGAAG
Actb-R*	*Actb*	TACGTACATGGCTGGGGTGTTGAAGGTCTC
Ahed-F	*Ahed*	AGCAAGACATTACCAATGGAA
Ahed-R	*Ahed*	CTTATGTTTTCGAGATTCACGG
Gata2-F†	*Gata2*	GGCTCTCCTGGTGTCTCTTACTCT
Gata2-R†	*Gata2*	GTCCACTACTGTGTCTTGGGAAC
Lmo2-F†	*Lmo2*	GAGAGACTATCTCAGGCTTTTTGG
Lmo2-R†	*Lmo2*	TTGAAACACTCCAGGTGATACACT
Oct3/4-F*	*Oct3/4*	CCTGGAATCGGACCAGGCTCAGAGGTATTG
Oct3/4-R*	*Oct3/4*	ATTGTTGTCGGCTTCCTCCACCCACTTCTC
p21-F‡	*p21*	ACAAGAGGCCCAGTACTTCC
p21-R‡	*p21*	GAAGACCAATCTGCGCTTGG
Runx1-F†	*Runx1*	CTCCGTGCTACCCACTCACT
Runx1-R†	*Runx1*	ATGACGGTGACCAGAGTGC
Tal1-F	*Tal1*	GAGATGGAGATTTCTGATGGTCCT
Tal1-R	*Tal1*	CGGAGGATCTCATTCTTGCTTAGT

*: Horie et al. 2011
†: Li et al. 2011
‡: Tokunaga et al. 2010

Supplementary Table 4

Supplementary Table 4. p21 (*Cdkn1a*) expression features of haematopoietic cells derived from FLs and adult BMs of control or *Ahed*-deficient mice.

Gene name	Normalised count							
	Ahed cKO BM HSPCs	*Ahed* cKO BM erythroblasts	Control BM HSPCs	Control BM erythroblasts	*Ahed* cKO FL HSPCs	*Ahed* cKO FL erythroblasts	Control FL HSPCs	Control FL erythroblasts
Cdkn1a	2,193	1,328	775	56	284	6,092	232	51

FL: foetal liver
BM: bone marrow
cKO: conditional knockout
HSPCs: haematopoietic stem/progenitor cells

Supplementary Table 5

Supplementary Table 5. Aberrant splicing events detected by Leafcutter.

Rank	Gene	Chromosome	N	q	Annotation
1	*4933407K13Rik*	*X*	20	9.72E-17	cryptic
2	–	*2*	61	1.15E-14	annotated
3	*Gbp4*	*5*	25	5.51E-13	cryptic
4	*Gas5*	*1*	7	2.03E-13	cryptic
5	*Slfn8*	*11*	17	5.26E-10	cryptic
6	*Tmem181b-ps*	*17*	13	4.24E-09	cryptic
7	*Ahed*	*9*	3	4.24E-09	cryptic
8	*9930111J21Rik1*	*11*	12	8.75E-09	cryptic
9	*Oas1g*	*5*	5	8.76E-09	cryptic
10	*Ppm1g*	*5*	6	1.30E-07	cryptic
11	*Arhgef1*	*7*	8	2.35E-07	cryptic
12	*Shisa5*	*9*	4	2.35E-07	cryptic
13	*Ewsr1*	*11*	4	0.00000122	cryptic
14	*Mx1*	*16*	25	0.00000318	cryptic
15	*Smc5*	*19*	6	0.00000318	cryptic
16	*Gna13*	*11*	6	0.00000583	cryptic
17	*Snapin*	*3*	6	0.00000642	cryptic
18	*Ate1*	*7*	6	0.0000094	cryptic
19	*Ythdf2*	*4*	3	0.0000108	cryptic
20	*Tmem134*	*19*	16	0.0000137	cryptic
21	*Gas5*	*16*	4	0.0000139	cryptic
22	–	*15*	21	0.0000161	annotated
23	*Ifi213*	*1*	9	0.0000165	cryptic
24	*Nt5c3*	*6*	14	0.0000168	cryptic
25	*Mis18a*	*16*	3	0.000019	cryptic
26	*Sel1l*	*12*	4	0.00004	cryptic
27	*Oass1g*	*5*	8	0.0000468	cryptic
28	*Dnal1*	*12*	3	0.0000475	cryptic
29	*Gm46430*	*13*	31	0.0000492	cryptic
30	–	*10*	2	0.0000815	annotated
31	*Ermard*	*17*	5	0.000129	cryptic
32	*Zfp60*	*7*	3	0.000129	cryptic
…					
111	*PU.1*	*2*	2	0.00327	cryptic

*Clusters are initially ranked in the cluster results table by adjusted P value.

Supplementary Table 6

Supplementary Table 6. Primers used for detecting the presence of aberrant transcripts.

Primer Name	Sequence (5'-3')	exon size(bp)	intro size(bp)
PU.1 (Spi-1) Forward 1	ACATGGAGCTGGAACAGATGCACGTCCTCG	121	920
PU.1 (Spi-1) Reverse 1	GCTGGGGACAAGGTTTGATAAGGGAAGCAC		
PU.1 (Spi-1) Forward 2	GTGCTTCCCTTATCAAACCTTGTCCCCAGC	246	920
PU.1 (Spi-1) Reverse 2	GAACTGGAAGGTACCTTTGTCCTTGTCCAC		
Zfp60 Forward	GGAGGAAGCCTGAAGAAGAATGGCCAACTC	82	1394
Zfp60 Reverse	AACAGCCACATCCCTAAATGTCACTGACCC		
Ythdf2 Forward 1	GGCCTTGCCTGTGGGCTCAAGTAAGGTTCG	103	703
Ythdf2 Reverse 1	GACCAAAAGGTCAAGGAAACAAAG		
Ythdf2 Forward 2	CTTTGTTTCCTTGACCTTTTGGTC	77	543
Ythdf2 Reverse 2	CGTTCCCCGTGAGGATCCGAGAGCCATGTC		
Sel1l Forward 1	CAGAAGCCAGGCGGTAATGAATAAATGCGG	144	758
Sel1l Reverse 1	ACCCCAGAGCTTTACTGCATTGGAACAGGG		
Sel1l Forward 2	AGGCGGCCCTGTTCCAATGCAGTAAAGCTC	168	488
Sel1l Reverse 2	AGGATGAGGACTACAATGCTGCAGTGGTCC		
Sel1l Forward 3	TGGACCACTGCAGCATTGTAGTCCTCATCC	198	824
Sel1l Reverse 3	TCTGGCTTCTCAAGGAGGCCATATCTTGGC		
Sel1l Forward 4	GAAAGCCAAGATATGGCCTCCTTGAGAAGC	158	455
Sel1l Reverse 4	ATTTCCAGAAAGCTGCTGAGCAAGGCTGGG		
Ythdf2 Forward 1	GGCCTTGCCTGTGGGCTCAAGTAAGGTTCG	156	1199
Ythdf2 Reverse 2	CGTTCCCCGTGAGGATCCGAGAGCCATGTC		
Sel1l Forward 1	CAGAAGCCAGGCGGTAATGAATAAATGCGG	276	1070
Sel1l Reverse 2	AGGATGAGGACTACAATGCTGCAGTGGTCC		
Sel1l Forward 2	AGGCGGCCCTGTTCCAATGCAGTAAAGCTC	355	1241
Sel1l Reverse 3	TCTGGCTTCTCAAGGAGGCCATATCTTGGC		

Supplementary Table 7

Supplementary Table 7. Primers used for mouse genotyping.

Primer Name	Usage	Sequence (5'-3')
f1	Genotyping of *Ahed* KO mice	AAGACTCCAGGGATGGCTAGAGGTAAAT
r1	Genotyping of *Ahed* KO mice	TGCTGAGCATGGTGTGTCATGTCTTTGG
r2	Genotyping of *Ahed* KO mice	GGTAAGGTCTCTGAGGTTTTGCAGTTTC
f2	Genotyping of *Ahed* KO and cKO mice	CCTACAGTTGTTAAGAGAACAAATCCCTGA
r3	Genotyping of *Ahed* KO and cKO mice	GAGTCAGAGACCAGCCTGACCTACATAGT
r4	Genotyping of *Ahed* KO and cKO mice	ATATTGGTAAGGTCTCTGAGGTTTTGCAGT
q1	Genotyping of *Ahed* cKO mice	TATGACTGACTGAATTAATGATAAGACTCC
q2	Genotyping of *Ahed* cKO mice and evaluation of the *Vav1-cre* efficiency	TTTACTGTGTTGGCAATATTTTGTATGATG
q3	Evaluation of the *Vav1-cre* efficiency	TGACACACCCATATACATTATAACTTCG
q4	Evaluation of the *Vav1-cre* efficiency	TGGAGAAGGCCGGGGTAAGTG
q5	Evaluation of the *Vav1-cre* efficiency	GCCTGTTTAAGGGAAAGAAGTTGAT

KO: knockout
cKO: conditional knockout

Supplementary Table 8. (a) Primers used for genotyping of ESCs (b) Primers used for vector construction.

a

Primer Name	Sequence (5'-3')
p1	AAAGATATAAACACGAAGATTTGAAGG
p2	TAGCTACACATGTGAATATCGTTGTCT
p3	CGATCAGAAACTTCTCGACAGAC
p4	TGTCATACTTATCCTGTCCCTTTTT

b

Primer Name	Sequence (5'-3')
AHED-F1	GAGAGTCGACCACCATGTCCCGGGTTCCGTTGGGGAAAG
AHED-R1	TCTCGAATTCTTAGTCATCCTCCGAGCTCTCAGC
AHED-F2	GATCCACCGGTCGCCACCATGTCCCGGGTTCCGTTGGG
AHED-R2	GGGGCGGAATTCGTTTTAGTCATCCTCCGAGCTCTCAGCA
AHED-F3	GAATTGATCTCTCGAGCCACCATGTCCCGGGTTCCGTTGGG
AHED-R3	TGGCGACCGGTGGATCGTCATCCTCCGAGCTCTCAGCAGAC
FLAG-AHED-F	GAGAGTCGACCACCATGGACTACAAGGACGACGATGACAAGTCCC GGGTTCCGTTGGGGAAAGTC
EGFP-F1	GAATTGATCTCTCGAGCCACCATGGTGAGCAAGGGCGAGGA
EGFP-R1	GGCGACCGGTGGATCCTTGTACAGCTCGTCCATGCCGAGA
EGFP-F2	ATCCACCGGTCGCCACCATGGTGAGCAAGGGCGAGGAGCT
EGFP-R2	GGGGCGGAATTCGTTTTACTTGTACAGCTCGTCCATGCCG
EGFP-R3	GGTGGCGACCGGTGGATCCTTGTACAGCTCGTCCATGCCG
EGFP-R4	GGGGCGGAATTCGTTTTACTTGTACAGCTCGTCCATGCCGAGA
AHED-del-F1	GAATCTCGAAAACCCATGCCTGACCCTGAGGTACAGGAGA
AHED-del-R1	GGGTTTTCGAGATTCACGGGCAGATGATTTTACCTGGGGA
AHED-del-F2	CCACCGGTCGCCACCCATAAGAAGTCAAAGAAATCCCATA
AHED-del-R2	GGGGCGGAATTCGTTTTAGACATGGACACTCTTCTCTTTCTTT
AHED-del-F3	GAATCTCGAAAAAAGCAACCACACAAGAGCAAGAAAAAGTCCAGG
AHED-del-R3	CTTTTTTCGAGATTCACGGGCAGATGATTTTACCTGGGGA
AHED-del-F4	GAAAAGGCCCATGCCTGACCCTGAGGTACAGGAGAGGACA
AHED-del-R4	GGCATGGGCCTTTTCCTAGTACTAGAAGCTCGAGTTTCCTCTG

Supplementary Table 9. Primers used for gene targeting in ESCs.

Primer Name	Sequence (5'-3')
Long-arm-F	GAGCTCGTTTAAACGGCGCGCCGTAAGTCAGACTTTGAGTTGGGTATGGTGG
Long-arm-LoxP-R	GTATAGCATACATTATACGAAGTTATAATGTATATGGGTGTGCCAGTGCACACTTG
Long-arm-LoxP-F	GTATAATGTATGCTATACGAAGTTATCACACATCATACAAAATATTGCCAACACAG
Long-arm-R	ACTTCGGAATTGTTAATTAAACTATTCTAATGGTGCCTGCTTTGCTTTCC
Short-arm-F	CACTAGTTCTAGAGCGGCCGCTTACAAAGAAACTGCAAAACCTCAGAGACC
Short-arm-R	CACTGCTCGACATTTAAATGCATTTCCTGAGGGCACACAGTAAACCATG
PGK-L1	GTTGGCGCCTACCGGTGGATGTGGAATGTG
Short-1R	AGAATGTCCCGGGTTCCGTTGGGGAAAGTC
Lox-upper	TATGACTGACTGAATTAATGATAAGACTCC
Lox-lower	TTTACTGTGTTGGCAATATTTTGTATGATG
Lox-lower-2	AACAACAAAACCCAAAGCAAAAAGAAAACC
FRT-upper	GCAGAGACACACTGATCGCCTTATTAGT
FRT-lower	ATCTGGACGTAAACTCCTCTTCAGACCT
FRT-lower-2	GGTAAGGTCTCTGAGGTTTTGCAGTTTC
Long-arm-check-F	GCACTCTGGGTAAATTATTAACATCTGAAC
Long-arm-check-R	GGCCGCTCTAGAACTAGTGGATCCACC
5-external-F	AGAGCTGATCGTGGAAATGGGAGAGACAGG
5-external-R	TGCCCTATCCCTTGATTATGAGCAGGTGCC
3-external-F	CCCGGGACATTCTTTAATCTGAAATGAACC
3-external-R	ACCCTCACTCATCCTAACTGGAAGAGCATG
Internal-F	GGTAGCCAACGCTATGTCCTGATAG
Internal-R	GTGGAGAGGCTATTCGGCTATGACT
