# Supplementary material for: A newly identified gene Ahed plays essential roles in murine haematopoiesis
Source: Nat Commun. 2024 Jun 25;15:5090. doi: 10.1038/s41467-024-49252-7 (PMC11199565; doi:10.1038/s41467-024-49252-7)
Supplement: Supplementary file 3 — Description of Additional Supplementary Files [file 41467_2024_49252_MOESM3_ESM.pdf]

<u>Description of Additional Supplementary Files</u>

Supplementary Data 1 – Listing of established homozygous mutant ES cells
