# Supplementary material for: A newly identified gene Ahed plays essential roles in murine haematopoiesis
Source: Nat Commun. 2024 Jun 25;15:5090. doi: 10.1038/s41467-024-49252-7 (PMC11199565; doi:10.1038/s41467-024-49252-7)
Supplement: Supplementary file 4 — Supplementary Data 1 [file 41467_2024_49252_MOESM4_ESM.pdf]

Homozygous ES cells

Vector Name	Vector Type	Clone Name	IGTC-Resistered ID	Gene Symbol	Gene Name
pCMT-SAhygpA-NP22	Retrovirus	K14G12	AyuK14G12	Nr5a2	nuclear receptor subfamily 5, group A, member 2
pCMT-SAhygpA-NP21	Retrovirus	K13F05	AyuK13F05	Phf20	PHD finger protein 20
pCMT-SAhygpA-NP21	Retrovirus	K8C12	AyuK8C12	Gm561	gene model 561, (NCBI)
pCMT-SAhygpA-NP21	Retrovirus	K8E11	AyuK8E11	Ube2c	ubiquitin-conjugating enzyme E2C
pCMT-SAhygpA-NP21	Retrovirus	K12C11	AyuK12C11	Ctdspl2	CTD (carboxy-terminal domain, RNA polymerase II, polypeptide A) small phosphatase like 2
pCMT-SAhygpA-NP22	Retrovirus	K14C04	AyuK14C04	Fubp3	far upstream element (FUSE) binding protein 3
pCMT-SAhygpA-NP22	Retrovirus	K14G01	AyuK14G01	Cdca7	cell division cycle associated 7
pCMT-SAhygpA-NP22	Retrovirus	K16A10	AyuK16A10	Fmnl2	formin-like 2
pT2F2-SAhygpA-NP21	Tol2	K19F03	AyuK19F03	Nmt2	N-myristoyltransferase 2
pCMT-SAhygpA-NP21	Retrovirus	K11E04	AyuK11E04	Csnk2a1	casein kinase 2, alpha 1 polypeptide
pCMT-SAhygpA-NP22	Retrovirus	K15G02	AyuK15G02	Kpna4	karyopherin (importin) alpha 4
pCMT-SAhygpA-NP21	Retrovirus	K6F01	AyuK6F01	Gpatch4	G patch domain containing 4
pCMT-SAhygpA-NP21	Retrovirus	K6D12	AyuK6D12	Smg5	Smg-5 homolog, nonsense mediated mRNA decay factor (C. elegans)
pCMT-SAhygpA-NP21	Retrovirus	K8B01	AyuK8B01	Ilf2	interleukin enhancer binding factor 2
pCMT-SAhygpA-NP21	Retrovirus	K10A10	AyuK10A10	Cdc42se1	CDC42 small effector 1
pCMT-SAhygpA-NP22	Retrovirus	K15A08	AyuK15A08	Anp32e	acidic (leucine-rich) nuclear phosphoprotein 32 family, member E
pCMT-SAhygpA-NP21	Retrovirus	K12C03	AyuK12C03	Fryl	furry homolog-like (Drosophila)
pCMT-SAhygpA-NP21	Retrovirus	K6B03	AyuK6B03	G3bp2	GTPase activating protein (SH3 domain) binding protein 2
pT2F2-SAhygpA-NP21	Tol2	K17E05	AyuK17E05	Ptpn11	protein tyrosine phosphatase, non-receptor type 11
pCMT-SAhygpA-NP21	Retrovirus	K7G11	AyuK7G11	Rsrc2	arginine/serine-rich coiled-coil 2
pCMT-SAhygpA-NP22	Retrovirus	K14E01	AyuK14E01	Kntc1	kinetochore associated 1
pCMT-SAhygpA-NP22	Retrovirus	K16C10	AyuK16C10	Gnb2	guanine nucleotide binding protein, beta 2
pCMT-SAhygpA-NP22	Retrovirus	K16E07	AyuK16E07	Lrch4	leucine-rich repeats and calponin homology (CH) domain containing 4
pT2F2-SAhygpA-NP21	Tol2	K18C02	AyuK18C02	1110007L15Rik	RIKEN cDNA 1110007L15 gene
pT2F2-SAhygpA-NP21	Tol2	K17A12	AyuK17A12	Lnx2	ligand of numb-protein X 2
pCMT-SAhygpA-NP21	Retrovirus	K10H04	AyuK10H04	Cbx3	chromobox homolog 3 (Drosophila HP1 gamma)
pCMT-SAhygpA-NP21	Retrovirus	K6E04	AyuK6E04	Armet	arginine-rich, mutated in early stage tumors
pCMT-SAhygpA-NP21	Retrovirus	K7G08	AyuK7G08	Rbm5	RNA binding motif protein 5
pCMT-SAhygpA-NP21	Retrovirus	K8C06	AyuK8C06	Cnot10	CCR4-NOT transcription complex, subunit 10
pCMT-SAhygpA-NP21	Retrovirus	K8G11	AyuK8G11	Nedd4	neural precursor cell expressed, developmentally down-regulted gene 4
pCMT-SAhygpA-NP21	Retrovirus	K9E04	AyuK9E04	AU019823	expressed sequence AU019823
pCMT-SAhygpA-NP21	Retrovirus	K9F02	AyuK9F02	Pml	promyelocytic leukemia
pCMT-SAhygpA-NP21	Retrovirus	K10C05	AyuK10C05	Map2k1	mitogen activated protein kinase kinase 1
pCMT-SAhygpA-NP21	Retrovirus	K12C12	AyuK12C12	Igsf9b	immunoglobulin superfamily, member 9B
pCMT-SAhygpA-NP21	Retrovirus	K13G06	AyuK13G06	BC024479	cDNA sequence BC024479
pCMT-SAhygpA-NP22	Retrovirus	K15E07	AyuK15E07	Leo1	Leo1, Paf1/RNA polymerase II complex component, homolog (S. cerevisiae)
pCMT-SAhygpA-NP22	Retrovirus	K16C12	AyuK16C12	Trim71	tripartite motif-containing 71
pT2F2-SAhygpA-NP21	Tol2	K18E01	AyuK18E01	Ibtk	inhibitor of Bruton agammaglobulinemia tyrosine kinase
pCMT-SAhygpA-NP21	Retrovirus	K6H07	AyuK6H07	Cbx1	chromobox homolog 1 (Drosophila HP1 beta)
pCMT-SAhygpA-NP21	Retrovirus	K6B09	AyuK6B09	Tbrg4	transforming growth factor beta regulated gene 4
pCMT-SAhygpA-NP21	Retrovirus	K8A06	AyuK8A06	Nmt1	N-myristoyltransferase 1
pCMT-SAhygpA-NP22	Retrovirus	K14E11	AyuK14E11	Mllt6	myeloid/lymphoid or mixed lineage-leukemia translocation to 6 homolog (Drosophila)
pCMT-SAhygpA-NP22	Retrovirus	K15H03	AyuK15H03	Psmc3ip	proteasome (prosome, macropain) 26S subunit, ATPase 3, interacting protein
pT2F2-SAhygpA-NP21	Tol2	K17B02	AyuK17B02	Tada2l	transcriptional adaptor 2 (ADA2 homolog, yeast)-like
pT2F2-SAhygpA-NP21	Tol2	K18B12	AyuK18B12	Epn2	epsin 2
pCMT-SAhygpA-NP21	Retrovirus	K13E11	AyuK13E11	Cbx5	chromobox homolog 5 (Drosophila HP1a)
pCMT-SAhygpA-NP22	Retrovirus	K16C06	AyuK16C06	Dgcr8	DiGeorge syndrome critical region gene 8
pCMT-SAhygpA-NP21	Retrovirus	K7C09	AyuK7C09	Axin1	axin 1
pCMT-SAhygpA-NP21	Retrovirus	K9F06	AyuK9F06	Ddx11	DEAD/H (Asp-Glu-Ala-Asp/His) box polypeptide 11 (CHL1-like helicase homolog, S. cerevisiae)
pCMT-SAhygpA-NP21	Retrovirus	K13G03	AyuK13G03	Zfp206	zinc finger protein 206
pT2F2-SAhygpA-NP21-Rev	Tol2	K20B09	AyuK20B09	Srpk1	serine/arginine-rich protein specific kinase 1
pCMT-SAhygpA-NP21	Retrovirus	K11E11	AyuK11E11	Wdr4	WD repeat domain 4

pCMT-SAhygpA-NP22	Retrovirus	K14A05	AyuK14A05	Trerf1	transcriptional regulating factor 1
pCMT-SAhygpA-NP21	Retrovirus	K12E07	AyuK12E07	Trip10	thyroid hormone receptor interactor 10
pT2F2-SAhygpA-NP21	Tol2	K18D01	AyuK18D01	Birc6	baculoviral IAP repeat-containing 6
pCMT-SAhygpA-NP21	Retrovirus	K12A07	AyuK12A07	Saps3	SAPS domain family, member 3
pT2F2-SAhygpA-NP21	Tol2	K19F08	AyuK19F08	Tex19	testis expressed gene 19
pCMT-SAhygpA-NP21	Retrovirus	K7G10	AyuK7G10	Zfp280b	zinc finger protein 280b
pCMT-SAhygpA-NP21	Retrovirus	K13A11	AyuK13A11	Rap1b	RAS related protein 1b
pCMT-SAhygpA-NP21	Retrovirus	K8D01	AyuK8D01	Eno1	enolase 1, alpha non-neuron
pCMT-SAhygpA-NP21	Retrovirus	K12E12	AyuK12E12	Thrap3	thyroid hormone receptor associated protein 3
pT2F2-SAhygpA-NP21	Tol2	K20B04	AyuK20B04	Tjp2	tight junction protein 2
pCMT-SAhygpA-NP21	Retrovirus	K6A05	AyuK6A05	Ranbp1	RAN binding protein 1
pT2F2-SAhygpA-NP21	Tol2	K18B07	AyuK18B07	Zfp518	zinc finger protein 518
pCMT-SAhygpA-NP22	Retrovirus	K14E08	AyuK14E08	Pspc1	paraspeckle protein 1
pCMT-SAhygpA-NP21	Retrovirus	K10H01	AyuK10H01	Mapk1ip1l	mitogen-activated protein kinase 1 interacting protein 1-like
pCMT-SAhygpA-NP21	Retrovirus	K6F11	AyuK6F11	Rnf44	ring finger protein 44
pCMT-SAhygpA-NP21	Retrovirus	K8F07	AyuK8F07	Slc39a14	solute carrier family 39 (zinc transporter), member 14
pCMT-SAhygpA-NP21	Retrovirus	K7D01	AyuK7D01	Cfdp1	craniofacial development protein 1
pCMT-SAhygpA-NP21	Retrovirus	K10C08	AyuK10C08	Gatad2a	GATA zinc finger domain containing 2A
pCMT-SAhygpA-NP22	Retrovirus	K16E08	AyuK16E08	Rbpms	RNA binding protein gene with multiple splicing
pCMT-SAhygpA-NP21	Retrovirus	K6C07	AyuK6C07	5830457O10Rik	RIKEN cDNA 5830457O10 gene
pCMT-SAhygpA-NP21	Retrovirus	K5F06	AyuK5F06	Eapp	E2F-associated phosphoprotein
pCMT-SAhygpA-NP21	Retrovirus	K11C02	AyuK11C02	Ahsa1	AHA1, activator of heat shock protein ATPase homolog 1 (yeast)
pCMT-SAhygpA-NP21	Retrovirus	K12A12	AyuK12A12	Mipol1	mirror-image polydactyly gene 1 homolog (human)
pT2F2-SAhygpA-NP21	Tol2	K17G03	AyuK17G03	Gtf2h1	general transcription factor II H, polypeptide 1
pT2F2-SAhygpA-NP21	Tol2	K19G11	AyuK19G11	BC057627	cDNA sequence BC057627
pCMT-SAhygpA-NP21	Retrovirus	K10D10	AyuK10D10	Ppp2r2d	protein phosphatase 2, regulatory subunit B, delta isoform
pCMT-SAhygpA-NP21	Retrovirus	K11D09	AyuK11D09	Zfp568	zinc finger protein 568
pCMT-SAhygpA-NP22	Retrovirus	K15F02	AyuK15F02	Deaf1	deformed epidermal autoregulatory factor 1 (Drosophila)
pCMT-SAhygpA-NP21	Retrovirus	K8G02	AyuK8G02	Nosip	nitric oxide synthase interacting protein
pT2F2-SAhygpA-NP21	Tol2	K19B07	AyuK19B07	5930416I19Rik	RIKEN cDNA 5930416I19 gene
pT2F2-SAhygpA-NP21	Tol2	K17A06	AyuK17A06	Trim6	tripartite motif protein 6
pCMT-SAhygpA-NP21	Retrovirus	K6C01	AyuK6C01	Jagn1	jagunal homolog 1 (Drosophila)
pCMT-SAhygpA-NP21	Retrovirus	K7H06	AyuK7H06	Cnbp	cellular nucleic acid binding protein
pCMT-SAhygpA-NP21	Retrovirus	K8D05	AyuK8D05	Ccdc77	coiled-coil domain containing 77
pCMT-SAhygpA-NP21	Retrovirus	K12E09	AyuK12E09	Rad51ap1	RAD51 associated protein 1
pCMT-SAhygpA-NP22	Retrovirus	K15G06	AyuK15G06	Capza2	capping protein (actin filament) muscle Z-line, alpha 2
pT2F2-SAhygpA-NP21	Tol2	K18B08	AyuK18B08	Setd5	SET domain containing 5
pT2F2-SAhygpA-NP21	Tol2	K18E04	AyuK18E04	Thumpd3	THUMP domain containing 3
pCMT-SAhygpA-NP21	Retrovirus	K7A07	AyuK7A07	Cxxc5	CXXC finger 5
pCMT-SAhygpA-NP21	Retrovirus	K9B05	AyuK9B05	Zfp532	zinc finger protein 532
pCMT-SAhygpA-NP21	Retrovirus	K7B02	AyuK7B02	Avpi1	arginine vasopressin-induced 1
pCMT-SAhygpA-NP21	Retrovirus	K12H05	AyuK12H05	Atad1	ATPase family, AAA domain containing 1
pCMT-SAhygpA-NP21	Retrovirus	K13F02	AyuK13F02	Tarbp2	TAR (HIV) RNA binding protein 2
pCMT-SAhygpA-NP21	Retrovirus	K6F07	AyuK6F07	Bat2d	BAT2 domain containing 1
pCMT-SAhygpA-NP21	Retrovirus	K7A06	AyuK7A06	Epb4.1l5	erythrocyte protein band 4.1-like 5
pCMT-SAhygpA-NP22	Retrovirus	K16H07	AyuK16H07	Nmnat2	nicotinamide nucleotide adenylyltransferase 2
pCMT-SAhygpA-NP21	Retrovirus	K11B06	AyuK11B06	Atf1	activating transcription factor 1
pCMT-SAhygpA-NP21	Retrovirus	K6G02	AyuK6G02	Eif4b	eukaryotic translation initiation factor 4B
pCMT-SAhygpA-NP21	Retrovirus	K10D02	AyuK10D02	Ubr5	ubiquitin protein ligase E3 component n-recognin 5
pCMT-SAhygpA-NP21	Retrovirus	K13C12	AyuK13C12	Jrk	jerky
pCMT-SAhygpA-NP21	Retrovirus	K12D06	AyuK12D06	Ccdc58	coiled-coil domain containing 58
pCMT-SAhygpA-NP21	Retrovirus	K12F03	AyuK12F03	Anks3	ankyrin repeat and sterile alpha motif domain containing 3
pT2F2-SAhygpA-NP21	Tol2	K17E10	AyuK17E10	Myh9	myosin, heavy polypeptide 9, non-muscle
pT2F2-SAhygpA-NP21	Tol2	K17H08	AyuK17H08	Pcbp2	poly(rC) binding protein 2
pT2F2-SAhygpA-NP21	Tol2	K19D05	AyuK19D05	Fam49b	family with sequence similarity 49, member B
pT2F2-SAhygpA-NP21	Tol2	K17C03	AyuK17C03	Hspbap1	Hspb associated protein 1
