# Supplementary material for: A newly identified gene Ahed plays essential roles in murine haematopoiesis
Source: Nat Commun. 2024 Jun 25;15:5090. doi: 10.1038/s41467-024-49252-7 (PMC11199565; doi:10.1038/s41467-024-49252-7)
Supplement: Supplementary file 5 — Reporting summary [file 41467_2024_49252_MOESM5_ESM.pdf]

nature portfolio

Corresponding author(s): Takafumi Yokota, MD, PhD

Last updated by author(s): May 10, 2024

Reporting Summary

Nature Portfolio wishes to improve the reproducibility of the work that we publish. This form provides structure for consistency and transparency in reporting. For further information on Nature Portfolio policies, see our Editorial Policies and the Editorial Policy Checklist.

Statistics

For all statistical analyses, confirm that the following items are present in the figure legend, table legend, main text, or Methods section.

n/a	Confirmed	
☐	☒	The exact sample size (*n*) for each experimental group/condition, given as a discrete number and unit of measurement
☒	☐	A statement on whether measurements were taken from distinct samples or whether the same sample was measured repeatedly
☐	☒	The statistical test(s) used AND whether they are one- or two-sided<br>*Only common tests should be described solely by name; describe more complex techniques in the Methods section.*
☒	☐	A description of all covariates tested
☒	☐	A description of any assumptions or corrections, such as tests of normality and adjustment for multiple comparisons
☐	☒	A full description of the statistical parameters including central tendency (e.g. means) or other basic estimates (e.g. regression coefficient) AND variation (e.g. standard deviation) or associated estimates of uncertainty (e.g. confidence intervals)
☐	☒	For null hypothesis testing, the test statistic (e.g. F, t, r) with confidence intervals, effect sizes, degrees of freedom and P value noted<br>*Give P values as exact values whenever suitable.*
☒	☐	For Bayesian analysis, information on the choice of priors and Markov chain Monte Carlo settings
☐	☒	For hierarchical and complex designs, identification of the appropriate level for tests and full reporting of outcomes
☐	☒	Estimates of effect sizes (e.g. Cohen's *d*, Pearson's *r*), indicating how they were calculated

Our web collection on statistics for biologists contains articles on many of the points above.

Software and code

Policy information about availability of computer code

Data collection	Flow cytometry<br>FACSAria IIIu (BD Biosciences)<br>FACSCanto II (BD Biosciences)<br><br>Cell Preparation<br>Cytospin preparations (Shandon)<br><br>Electroporation<br>Gene Pulser II (Bio-Rad)<br><br>Complete blood count analysis<br>VetScan HM2 hematology analyzer (Abaxis)<br><br>qPCR<br>ABI PRISM 7900 HT (Applied Biosystems Inc.)
Data analysis	Flow cytometry<br>Flow Jo software (v9.9.6 and 10.8.1) (FlowJo, LLC)<br><br>Image analysis<br>BBZ-X710 (KEYENCE)

Statistical analysis
Graphpad Prism 7 and 8 (GraphPad Software, Inc)

RNA-Seq analysis
Leafcutter(Li, et al., https://davidaknowles.github.io/leaf cutter/)
RTA v3.4.4 (llumina, http://samtools.sourceforge.net/)
TopHat v2.1.1 (JHU CCB, https://ccb.jhu.edu/software/tophat/ index.shtml)
Bowtie2 v2.2.8 (N/A, http://bowtie- bio.sourceforge.net/bowtie2/index. shtml)
SAMtools v0.1.18 (N/A, http://samtools.sourceforge.net/)
Cuffdiff v2.2.1 (N/A, http://cole-trapnell-lab.github.io/cufflinks/)
STAR (N/A, https://github.com/alexdobin/STAR)
sra-tools v2,.10.9 (N/A, https://github.com/ncbi/sra-tools)
FastQC v0.11.9 (N/A, https://www.bioinformatics.babraha m.ac.uk/projects/fastqc/)
MultiQC v0.10.1 (N/A, https://multiqc.info/docs/)
Trim Galore! v0.6.7 (N/A, https://www.bioinformatics.babraha m.ac.uk/projects/trim_galore/)
Salmon v1.4.0 (N/A, https://github.com/COMBINE- lab/salmon/releases)
tximport v1.6.0 (N/A, https://bioconductor.org/packages/r elease/bioc/html/tximport.html)
iDEP .94 (N/A, http://bioinformatics.sdstate.edu/id ep94/)
Enrichr (N/A, https://maayanlab.cloud/Enrichr/)

For manuscripts utilizing custom algorithms or software that are central to the research but not yet described in published literature, software must be made available to editors and reviewers. We strongly encourage code deposition in a community repository (e.g. GitHub). See the Nature Portfolio guidelines for submitting code & software for further information.

Data

Policy information about availability of data

All manuscripts must include a data availability statement. This statement should provide the following information, where applicable:

- Accession codes, unique identifiers, or web links for publicly available datasets
- A description of any restrictions on data availability
- For clinical datasets or third party data, please ensure that the statement adheres to our policy

All RNA-seq data were deposited at Gene Expression Omnibus (GEO) under accessions GSE218517 (RNA-seq on E14.5 FL or BM LSK CD48- cells) [https://www.ncbi.nlm.nih.gov/geo/query/acc.cgi?acc=GSE218517] and GSE218518 (RNA-seq on triplicate differentiated populations to detect alternative splicing events) [https://www.ncbi.nlm.nih.gov/geo/query/acc.cgi?acc=GSE218518]. Source data are provided with this paper. All other data that support the findings of this study are available from the lead contact upon reasonable request.

Research involving human participants, their data, or biological material

Policy information about studies with human participants or human data. See also policy information about sex, gender (identity/presentation), and sexual orientation and race, ethnicity and racism.

Reporting on sex and gender	N/A
Reporting on race, ethnicity, or other socially relevant groupings	N/A
Population characteristics	N/A
Recruitment	N/A
Ethics oversight	N/A

Note that full information on the approval of the study protocol must also be provided in the manuscript.

Field-specific reporting

Please select the one below that is the best fit for your research. If you are not sure, read the appropriate sections before making your selection.

☒ Life sciences ☐ Behavioural & social sciences ☐ Ecological, evolutionary & environmental sciences

For a reference copy of the document with all sections, see nature.com/documents/nr-reporting-summary-flat.pdf

Life sciences study design

All studies must disclose on these points even when the disclosure is negative.

Sample size	The sample size was not predetermined because it is difficult to predict in advance the number of embryos of a particular genotype in each

Sample size	litter. However, the number of timed matings was adjusted to minimise the number of embryos to be analysed while still allowing for sufficient statistical analysis.
Data exclusions	Fig. 5c, complete blood count data; one mouse in the R26CE Ahedfl/fl group could not have an accurate platelet count due to blood coagulation, so this one was excluded from the data analysis for platelet count only.
Replication	Experiments were done in more that triplicate and each experiment was repeated for two or more repeats to confirm reproducibility.
Randomization	Samples and animals were assigned to group randomly distributed in different groups before treatments.
Blinding	In order to analyse specific genotypes efficiently and reduce the number of mice to be analysed, blinding was not performed. However, all experiments followed the same workflow.

Reporting for specific materials, systems and methods

We require information from authors about some types of materials, experimental systems and methods used in many studies. Here, indicate whether each material, system or method listed is relevant to your study. If you are not sure if a list item applies to your research, read the appropriate section before selecting a response.

Materials & experimental systems

n/a	Involved in the study
☐	☒ Antibodies
☐	☒ Eukaryotic cell lines
☒	☐ Palaeontology and archaeology
☐	☒ Animals and other organisms
☒	☐ Clinical data
☒	☐ Dual use research of concern
☒	☐ Plants

Methods

n/a	Involved in the study
☒	☐ ChIP-seq
☐	☒ Flow cytometry
☒	☐ MRI-based neuroimaging

Antibodies

Antibodies used

Primary antibodies used in this paper were as follows;

Anti-mouse CD3e Antibody (clone: 145-2C11), 1:50, BioLegend, Cat# 100328; RRID: AB_893318, FCM
Anti-mouse CD11b Antibody (clone: M1/70), 1:50, BD Biosciences, Cat# 553310; RRID: AB_396679, FCM
Anti-mouse CD11b Antibody (clone: M1/70), 1:50, BioLegend, Cat# 101206; RRID: AB_312789, FCM
Anti-mouse CD11b Antibody (clone: M1/70), 1:50, BD Biosciences, Cat# 553311; RRID: AB_396680, FCM
Anti-mouse CD11b Antibody (clone: M1/70), 1:50, BD Biosciences, Cat# 553312; RRID: AB_398535, FCM
Anti-mouse CD11b Antibody (clone: M1/70), 1:50, BioLegend, Cat# 101228; RRID: AB_893232, FCM
Anti-mouse CD16/32 Antibody (clone: 93), 1:50, BioLegend, Cat# 101302; RRID: AB_312801, FCM
Anti-mouse CD16/32 Antibody (clone: 93), 1:50, BioLegend, Cat# 101308; RRID: AB_312807, FCM
Anti-mouse CD19 Antibody (clone: 6D5), 1:50, BioLegend, Cat# 152410; RRID: AB_2629839, FCM
Anti-mouse CD31 Antibody (clone: MEC13.3), 1:500, BD Biosciences, Cat# 553373; RRID: AB_394819, IF
Anti-mouse CD31 Antibody (clone: MEC13.3), 1:50, BioLegend, Cat# 102406; RRID: AB_312901, FCM
Anti-mouse CD34 Antibody (clone: RAM34), 1:50, BD Biosciences, Cat# 553733; RRID: AB_1645242, FCM
Anti-mouse CD45 Antibody (clone: 30-F11), 1:100, BioLegend, Cat# 103106; RRID: AB_312971, FCM
Anti-mouse CD45R/B220 Antibody (clone: RA3-6B2), 1:50, BD Biosciences, Cat# 561878; RRID: AB_394619, FCM
Anti-mouse CD45R/B220 Antibody (clone: RA3-6B2), 1:50, BD Biosciences, Cat# 552771; RRID: AB_394457, FCM
Anti-mouse CD45R/B220 Antibody (clone: RA3-6B2), 1:50, BioLegend, Cat# 103236; RRID: AB_893354, FCM
Anti-mouse CD48 Antibody (clone: HM48-1), 1:50, BioLegend, Cat# 103404; RRID: AB_313019, FCM
Anti-mouse CD71 Antibody (clone: C2), 1:50, BD Biosciences, Cat# 553267; RRID: AB_394744, FCM
Anti-mouse CD71 Antibody (clone: RI7217), 1:50, BioLegend, Cat# 113808; RRID: AB_313569, FCM
Anti-mouse CD117 Antibody (clone: 2B8), 1:50, BioLegend, Cat# 105814; RRID: AB_313223, FCM
Anti-mouse CD127 Antibody (clone: A7R34), 1:50, BioLegend, Cat# 1937273; RRID: AB_135022, FCM
Anti-mouse CD127 Antibody (clone: A7R34), 1:50, BioLegend, Cat# 135024; RRID: AB_11218800, FCM
Anti-mouse CD135 Antibody (clone: A2F10), 1:50, BioLegend, Cat# 135308; RRID: AB_1953267, FCM
Anti-mouse CD150 Antibody (clone: TC15-12F12.2), 1:50, BioLegend, Cat# 115937; RRID: AB_2565962, FCM
Anti-mouse CD202b Antibody (clone: TEK4), 1:50, eBiosciences, Cat# 12-5987-82; RRID: AB_466100, FCM
Anti-mouse ESAM Antibody (clone: 1GB/ESAM), 1:50, BioLegend, Cat# 136207; RRID: AB_2101658, FCM
Anti-mouse Flk1 Antibody (clone: Avas12a1), 1:50, BD Biosciences, Cat# 555308; RRID: AB_395721, FCM
Anti-mouse Flk1 Antibody (clone: Avas12), 1:50, BioLegend, Cat# 136406; RRID: AB_2044067, FCM
Anti-mouse Gr-1 Antibody (RB6-8C5), 1:50, BioLegend, Cat# 108428; RRID: AB_893558, FCM
Anti-mouse Sca-1 Antibody (E13-161.7), 1:50, BD Biosciences, Cat# 553335; RRID: AB_394791, FCM
Anti-mouse Sca-1 Antibody (E13-161.7), 1:50, BioLegend, Cat# 108106; RRID: AB_313343, FCM
Anti-mouse Sca-1 Antibody (D7), 1:50, BioLegend, Cat# 108126; RRID: AB_10645327, FCM
Anti-mouse TER-119/erythroid cell Antibody (clone: TER119), 1:50, BD Biosciences, Cat# 553673; RRID: AB_394986, FCM
Anti-mouse TER-119/erythroid cell Antibody (clone: TER119), 1:50, BioLegend, Cat# 116228; RRID: AB_893636, FCM
Anti-mouse TER-119/erythroid cell Antibody (clone: TER119), 1:50, BioLegend, Cat# 116206; RRID: AB_313707, FCM

Donkey antibody to goat IgG Antibody (Donkey polyclonal), 1:50, Thermo Fisher, Cat# A-11055, IF
Donkey Antibody to rabbit IgG Antibody (Donkey polyclonal), Thermo Fisher, Cat# A-21206, IF
FLAG Antibody (clone: M2), Sigma-Aldrich, Cat# F3165, IF
Goat Antibody to mouse IgG Antibody (Goat polyclonal), Thermo Fisher, Cat# A-11029, IF

FCM: Flow cytometry
IF: Immunofluorescence

Validation

All antibodies are commercially available and were validated based on information provided by the supplier. Titration experiments were performed rior to the study.

Anti-mouse CD3e Antibody: https://www.biolegend.com/ja-jp/sean-tuckers-tests/percp-cyanine5-5-anti-mouse-cd3epsilon-antibody-4191
Anti-mouse CD11b Antibody: https://www.bdbiosciences.com/en-sg/products/reagents/flow-cytometry-reagents/research-reagents/single-color-antibodies-ruo/fitc-rat-anti-cd11b.557396
Anti-mouse CD11b Antibody: https://www.biolegend.com/ja-jp/products/fitc-anti-mouse-human-cd11b-antibody-347?GroupID=BLG10660
Anti-mouse CD11b Antibody: https://www.bdbiosciences.com/ja-jp/products/reagents/flow-cytometry-reagents/research-reagents/single-color-antibodies-ruo/pe-rat-anti-cd11b.553311
Anti-mouse CD11b Antibody: https://www.bdbiosciences.com/ja-jp/products/reagents/flow-cytometry-reagents/research-reagents/single-color-antibodies-ruo/apc-rat-anti-cd11b.553312
Anti-mouse CD11b Antibody: https://www.biolegend.com/ja-jp/products/percp-cyanine5-5-anti-mouse-human-cd11b-antibody-4257
Anti-mouse CD16/32 Antibody: https://www.biolegend.com/de-de/products/purified-anti-mouse-cd16-32-antibody-190?GroupID=BLG9237
Anti-mouse CD16/32 Antibody: https://www.biolegend.com/ja-jp/products/pe-anti-mouse-cd16-32-antibody-189?GroupID=BLG6831
Anti-mouse CD19 Antibody: https://www.biolegend.com/ja-jp/products/apc-anti-mouse-cd19-antibody-13680
Anti-mouse CD31 Antibody: https://www.bdbiosciences.com/ja-jp/products/reagents/flow-cytometry-reagents/research-reagents/single-color-antibodies-ruo/pe-rat-anti-mouse-cd31.553373
Anti-mouse CD31 Antibody: https://www.biolegend.com/ja-jp/products/fitc-anti-mouse-cd31-antibody-120?GroupID=BLG1566, FCM
Anti-mouse CD34 Antibody: https://www.bdbiosciences.com/ja-jp/products/reagents/flow-cytometry-reagents/research-reagents/single-color-antibodies-ruo/fitc-rat-anti-mouse-cd34.553733
Anti-mouse CD45 Antibody: https://www.biolegend.com/ja-jp/products/pe-anti-mouse-cd45-antibody-100
Anti-mouse CD45R/B220 Antibody: https://www.bdbiosciences.com/en-us/products/reagents/flow-cytometry-reagents/research-reagents/single-color-antibodies-ruo/pe-rat-anti-mouse-cd45r-b220.561878
Anti-mouse CD45R/B220 Antibody: https://www.bdbiosciences.com/en-us/products/reagents/flow-cytometry-reagents/research-reagents/single-color-antibodies-ruo/percp-cy-5-5-rat-anti-mouse-cd45r-b220.552771
Anti-mouse CD45R/B220 Antibody: https://www.biolegend.com/ja-jp/products/percp-cyanine5-5-anti-mouse-human-cd45r-b220-antibody-4267?GroupID=BLG6847
Anti-mouse CD48 Antibody: https://www.biolegend.com/ja-jp/products/fitc-anti-mouse-cd48-antibody-291
Anti-mouse CD71 Antibody: https://www.bdbiosciences.com/en-us/products/reagents/flow-cytometry-reagents/research-reagents/single-color-antibodies-ruo/pe-rat-anti-mouse-cd71.561937#!
Anti-mouse CD71 Antibody: https://www.biolegend.com/ja-jp/products/pe-anti-mouse-cd71-antibody-1631?GroupID=BLG4775
Anti-mouse CD117 Antibody: https://www.biolegend.com/ja-jp/products/pe-cyanine7-anti-mouse-cd117-c-kit-antibody-1900?GroupID=BLG1945
Anti-mouse CD127 Antibody: https://www.biolegend.com/ja-jp/products/percp-cyanine5-5-anti-mouse-cd127-il-7ralpha-antibody-6196?GroupID=BLG7953
Anti-mouse CD127 Antibody: https://www.biolegend.com/ja-jp/products/brilliant-violet-421-anti-mouse-cd127-il-7ralpha-antibody-7193
Anti-mouse CD135 Antibody: https://www.biolegend.com/de-de/products/biotin-anti-mouse-cd135-antibody-6270?GroupID=BLG7932
Anti-mouse CD150 Antibody: https://www.biolegend.com/ja-jp/products/brilliant-violet-785-anti-mouse-cd150-slam-antibody-12080?GroupID=BLG4784
Anti-mouse CD202b Antibody: https://www.thermofisher.com/antibody/product/CD202b-TIE2-Antibody-clone-TEK4-Monoclonal/12-5987-82
Anti-mouse ESAM Antibody: https://www.biolegend.com/ja-jp/products/apc-anti-mouse-esam-antibody-6528
Anti-mouse Flk1 Antibody: https://www.bdbiosciences.com/en-in/products/reagents/flow-cytometry-reagents/research-reagents/single-color-antibodies-ruo/pe-rat-anti-mouse-flk-1.555308
Anti-mouse Flk1 Antibody: https://www.biolegend.com/ja-jp/products/apc-anti-mouse-cd309-vegfr2-flk-1-antibody-6472?GroupID=BLG8270
Anti-mouse Gr-1 Antibody: https://www.biolegend.com/fr-lu/products/percp-cyanine5-5-anti-mouse-ly-6g-ly-6c-gr-1-antibody-4286
Anti-mouse Sca-1 Antibody: https://www.bdbiosciences.com/ja-jp/products/reagents/flow-cytometry-reagents/research-reagents/single-color-antibodies-ruo/fitc-rat-anti-mouse-ly-6a-e.553335
Anti-mouse Sca-1 Antibody: https://www.biolegend.com/ja-jp/products/fitc-anti-mouse-ly-6a-e-sca-1-antibody-21659?GroupID=ImportedGROUP1
Anti-mouse Sca-1 Antibody: https://www.biolegend.com/ja-jp/products/apc-cyanine7-anti-mouse-ly-6a-e-sca-1-antibody-6752
Anti-mouse TER-119/erythroid cell Antibody: https://www.bdbiosciences.com/ja-jp/products/reagents/flow-cytometry-reagents/research-reagents/single-color-antibodies-ruo/pe-rat-anti-mouse-ter-119-erythroid-cells.553673
Anti-mouse TER-119/erythroid cell Antibody: https://www.biolegend.com/ja-jp/products/percp-cyanine5-5-anti-mouse-ter-119-erythroid-cells-antibody-4292?GroupID=ImportedGROUP1
Anti-mouse TER-119/erythroid cell Antibody: https://www.biolegend.com/ja-jp/products/fitc-anti-mouse-ter-119-erythroid-cells-antibody-1865
Donkey antibody to goat IgG Antibody: https://www.thermofisher.com/antibody/product/Donkey-anti-Goat-IgG-H-L-Cross-Adsorbed-Secondary-Antibody-Polyclonal/A-11055
Donkey Antibody to rabbit IgG Antibody: https://www.thermofisher.com/antibody/product/Donkey-anti-Rabbit-IgG-H-L-Highly-Cross-Adsorbed-Secondary-Antibody-Polyclonal/A-21206
FLAG Antibody: https://www.sigmaaldrich.com/deepweb/assets/sigmaaldrich/product/documents/415/240/f3165dat-mk.pdf

Goat Antibody to mouse IgG Antibody: https://www.thermofisher.com/antibody/product/Goat-anti-Mouse-IgG-H-L-Highly-Cross-Adsorbed-Secondary-Antibody-Polyclonal/A-11029

Eukaryotic cell lines

Policy information about cell lines and Sex and Gender in Research

Cell line source(s)	KY1.1 (129S6/B6 F1 hybrid ESCs)<br>MS-5 (murine stromal cell line)<br>OP-9
Authentication	KY1.1; This manuscript, MS-5; https://cellbank.brc.riken.jp/cell_bank/CellInfo/?cellNo=RCB4680&lang=Ja, OP-9; https://cellbank.brc.riken.jp/cell_bank/CellInfo/?cellNo=RCB1124&lang=Ja.
Mycoplasma contamination	The cell line was tested negative for Mycoplasma contamination.
Commonly misidentified lines<br>(See ICLAC register)	There was no misidentified cell lines in this study.

Animals and other research organisms

Policy information about studies involving animals; ARRIVE guidelines recommended for reporting animal research, and Sex and Gender in Research

Laboratory animals	Mus musculus was used as the animal model. All mice used in this study were of C57BL/6J background, except for foster mothers (ICR). Males and females were used in this study. Embryos were analysed from E9.5 to 18.5 (E9.5, E10.5, E11.5, E12.5, E13,5, E14.5, E16.5, E18.5) and adult mice were collected at 6w-6mo. The strains used in this study are shown as bellow.<br><br>Mouse: C57BL/6J (Clea Japan)<br>Mouse: DBA/2 (Clea Japan)<br>Mouse: Ahed-floxed (this paper)<br>Mouse: B6.Cg-Commd10Tg(Vav1-cre)A2Kio/J (Vav1- cre) (The Jackson Laboratory)<br>Mouse: B6.129-Gt(ROSA)26Sortm1(cre/ERT2)Tyj/J (Rosa26-CreERT2) (The Jackson Laboratory)<br>Mouse: B6.Tie2-cre (The Jackson Laboratory)
Wild animals	This study did not use any wild animals.
Reporting on sex	Our research results do not apply to only one sex.
Field-collected samples	This study did not include field-collected samples.
Ethics oversight	This research complies with all relevant ethical regulations. All animal experiments were conducted in accordance with institutional guidelines and were approved by the Institutional Animal Care and Use Committee of Osaka University Graduate School of Medicine (Approval No. 30-096-013) and the Recombinant Gene Experiment Review Committee of Osaka University Graduate School of Medicine (Approval No. 04418). Our manuscript abides by the ARRIVE (Animal Research: Reporting of In Vivo Experiments) guidelines for reporting of animal experiments. All mice used in this study were maintained under specific pathogen-free conditions in an animal facility at Osaka University Graduate School of Medicine (Osaka, Japan). The mice involved in this study were housed under a 12-hour light/dark cycle, with a stable temperature between 21.5°C and 24.5°C and a relative humidity range of 45-65%. They were provided with a standard laboratory chow diet and had ad libitum access to water. All mice used in this study were euthanized under anesthesia.

Note that full information on the approval of the study protocol must also be provided in the manuscript.

Plants

Seed stocks	This study did not involve seed stocks.
Novel plant genotypes	N/A
Authentication	N/A

Flow Cytometry

Plots

Confirm that:

☒ The axis labels state the marker and fluorochrome used (e.g. CD4-FITC).

☒ The axis scales are clearly visible. Include numbers along axes only for bottom left plot of group (a 'group' is an analysis of identical markers).

☒ All plots are contour plots with outliers or pseudocolor plots.

☒ A numerical value for number of cells or percentage (with statistics) is provided.

Methodology

Sample preparation	Fetal liver (FL) was dissected and minced with 5mL syringe. Bone marrow (BM) cells were isolated by flushing the femurs and tibias with staining buffer (PBS supplemented with 3% FCS, 100 U mL-1 penicillin, and 100 μg mL-1 streptomycin) by using a needle and syringe. FL and BM cells were gently filtered through a nylon screen (70 mm) to obtain a single-cell suspension. For analyzing E10.5 embryos, single-cell suspensions were prepared by treating tissues with collagenase (0.125% in PBS/10% fetal calf serum (FCS)/1% penicillin/ streptomycin) for 1 h at 37 °C.
Instrument	FACSAria IIIu and FACSCanto II (BD Biosciences).
Software	BD FACSDiva software (BD Biosciences) or Flow Jo software version 9.9.6 and 10.8.1 (FlowJo,LLC).
Cell population abundance	Purity check was performed on a small fraction of sorted cells (>90-95%).
Gating strategy	Combinations of cell-surface markers were used to identify the following populations: LSK, Lineage (CD11b, Gr1, CD3ε, CD45R/B220, and Ter119)– Sca1+ c-kitHi; LT-HSC, LSK CD150+ CD48- Flt3-; MPPs, LSK CD150- CD48- Flt3-; LMPPs, LSK Flt3+ IL-7Ra-; CLPs, Lin c-KitLo Sca-1-/Lo Flt3+ IL-7Ra+; CMPs, Lin- Sca1- c-Kit+ FCgRLo CD34Hi; GMPs, Lin- Sca1- c-Kit+ FCgRHi CD34Hi; MEPs, Lin- Sca1- c-Kit+ FCgRLo CD34Lo; S0, Ter119-CD71-; S1, Ter119-CD71+; S2, Ter119LoCD71+; S3, Ter119+CD71+; S4, Ter119- CD71+; haematopoietic cluster, Ter119-c-Kit+CD31+; and haemogenic endothelial cells, c-Kit+CD31+CD45- were stained as previously described. Doublets were excluded by forward scatter height/width (FSC-H/FSC-W) and side scatter height/width (SSC-H/SSC-W) profiles. Dead cells were excluded by 7-AAD staining.

☒ Tick this box to confirm that a figure exemplifying the gating strategy is provided in the Supplementary Information.
